# 2235. Characterization of the Adult Sepsis Treatment Guideline Utilization Within the Emergency Department at an Academic Medical Center

**DOI:** 10.1093/ofid/ofad500.1857

**Published:** 2023-11-27

**Authors:** Deanna M Berg, Christina Maguire, Amanda Binkley, Matthew Hinton, Sean Foster, Lauren Dutcher

**Affiliations:** Penn Presbyterian Medical Center, PHILADELPHIA, Pennsylvania; Penn Presbyterian Medical Center, PHILADELPHIA, Pennsylvania; Penn Presbyterian Medical Center, PHILADELPHIA, Pennsylvania; Penn Presbyterian Medical Center, PHILADELPHIA, Pennsylvania; Penn Presbyterian Medical Center, PHILADELPHIA, Pennsylvania; University of Pennsylvania Perelman School of Medicine, Philadelphia, Pennsylvania

## Abstract

**Background:**

The early initiation of antibiotics in patients with sepsis has been identified as the most effective intervention in reducing mortality. Screening tools have been developed for the early identification of sepsis. This study characterized Emergency Department (ED) patients initiated on antibiotics for suspected sepsis of urinary or abdominal sources to identify opportunities to improve institutional sepsis guidelines.

**Methods:**

A retrospective chart review was performed of patients initiated on empiric antibiotics in the ED for sepsis with a suspected source of urinary tract infection (UTI) or intraabdominal infection (IAI) from September 2021 to September 2022. Epic SlicerDicer, an electronic medical record (EMR) data extraction tool, identified ED patients with an order for broad-spectrum antibiotics. Patients less than 18 years or initiated on antibiotics prior to arrival were excluded. The EMR was utilized to assess the signs of organ dysfunction, antibiotic regimen, EMR-based sepsis pathway use, and patient-specific risk factors. Institutional guidelines defined antibiotic regimen appropriateness. Descriptive statistics were used to characterize the results.

**Figure:**
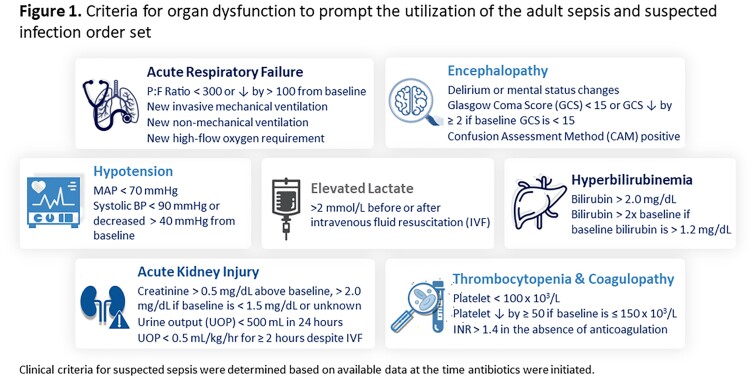

**Results:**

Among the 6869 patients screened, 190 patients were included. The provider initiated the sepsis pathway in 141 (74.2%) patients, and 21 (14.9%) were initiated on empiric antibiotics determined to be congruent with institutional guidelines. The most common deviation from institutional guidelines was the addition of anti-methicillin-resistant *Staphylococcus aureus* (MRSA) treatment. Anti-pseudomonal therapy was used in 165 (86.8%) patients and anti-MRSA coverage in 117 (61.6%) patients. Of patients with MRSA coverage, 36 (30.8%) had community-acquired infections. An organism was isolated in 113 (59.5%) patients, and Enterobacterales were most commonly isolated (65.5%). *P. aeruginosa* was isolated in 15 (7.9%) patients, and no patients had MRSA.

**Figure:**
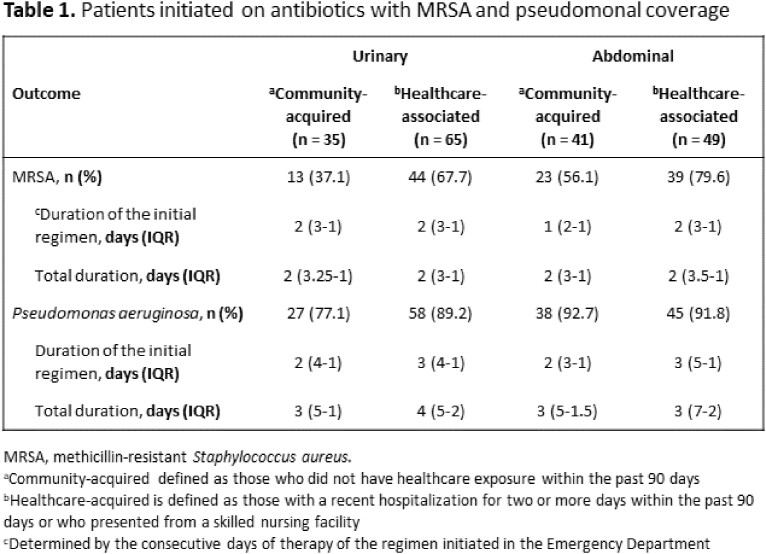

**Figure:**
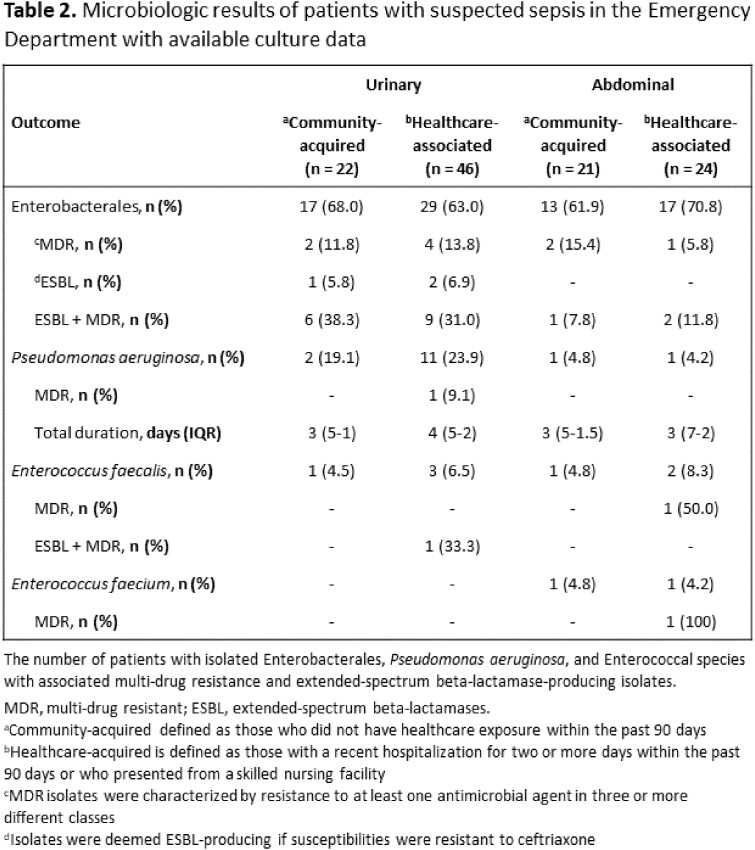

**Conclusion:**

Most antibiotic regimens used in ED patients for the treatment of sepsis with suspected urinary or abdominal sources of infection were contrary to institutional guidelines. This may be a result of an overestimation of MRSA or *P. aeruginosa* in community-acquired infections, highlighting an opportunity for education and stewardship.

**Disclosures:**

**Christina Maguire, PharmD**, Viiv: Advisor/Consultant **Amanda Binkley, PharmD, BCIDP, AAHIVP**, Viiv: Advisor/Consultant

